# Grapevine Responses to Heat Stress and Global Warming

**DOI:** 10.3390/plants9121754

**Published:** 2020-12-11

**Authors:** Xenophon Venios, Elias Korkas, Aspasia Nisiotou, Georgios Banilas

**Affiliations:** 1 Department of Wine, Vine and Beverage Sciences, University of West Attica, Ag. Spyridonos 28, 12243 Athens, Greece; xvenios@uniwa.gr (X.V.); elkorkas@uniwa.gr (E.K.); 2 Institute of Technology of Agricultural Products, Hellenic Agricultural Organization “Demeter”, Sofokli Venizelou 1, 14123 Lykovryssi, Greece; anisiotou.wi@nagref.gr

**Keywords:** global warming, grapevine, heat stress, molecular responses, phenology, viticulture, *Vitis*, wine

## Abstract

The potential effects of the forthcoming climate change include the rising of the average annual temperature and the accumulation of extreme weather events, like frequent and severe heatwaves, a phenomenon known as global warming. Temperature is an important environmental factor affecting almost all aspects of growth and development in plants. The grapevine (*Vitis* spp.) is quite sensitive to extreme temperatures. Over the current century, temperatures are projected to continue rising with negative impacts on viticulture. These consequences range from short-term effects on wine quality to long-term issues such as the suitability of certain varieties and the sustainability of viticulture in traditional wine regions. Many viticultural zones, particularly in Mediterranean climate regions, may not be suitable for growing winegrapes in the near future unless we develop heat-stress-adapted genotypes or identify and exploit stress-tolerant germplasm. Grapevines, like other plants, have developed strategies to maintain homeostasis and cope with high-temperature stress. These mechanisms include physiological adaptations and activation of signaling pathways and gene regulatory networks governing heat stress response and acquisition of thermotolerance. Here, we review the major impacts of global warming on grape phenology and viticulture and focus on the physiological and molecular responses of the grapevine to heat stress.

## 1. Introduction

Climate change is one of the biggest environmental challenges that humanity will face over the next few decades according to reports from the Intergovernmental Panel of Climate Change (http://www.ipcc.ch). The release of greenhouse gases, especially CO_2_, due to various anthropogenic activities is regarded as the main cause of climate change and in particular global warming [1,2]. During the 20th century, the concentration of CO_2_ has escalated from 280 to 400 ppm resulting in an average temperature rise of 0.5–1 °C. It is expected that the CO_2_ concentration will elevate further and the global average temperature will rise by 0.2–0.3 °C per decade, reaching values between 1.2 and 5.8 °C by the end of the twenty-first century [3]. Temperature records with emphasis on viticultural areas show that during the growing seasons from 1950 to 2000 the mean temperature has increased by about 1.6–1.8 °C in Europe and 1.2–1.4 °C worldwide [3,4]. Importantly, parallel to climate change, heatwaves are becoming more common and extreme high-temperature events more frequent.

Changes in grapevine phenology are regarded as one of the most unambiguous consequences of global warming. Recent studies show that temperature rise is highly correlated with an earlier onset of many growth stages in the grapevine [5,6]. Furthermore, the shortening of the duration of most phenological stages due to increased global temperature may adversely affect the composition and quality of grapes and thus of wine. All these changes highlight the need to take adaptation measures such as the relocation of vineyard cultivation to northern zones or higher altitude areas with lower average temperatures in order to maintain the quality of the final products to the desired level [7].

Plant growth and many developmental processes are strongly influenced by ambient temperature fluctuations. Each species has a preferred temperature range, which is represented by optimum, maximum and minimum values. Extreme temperatures are among the most significant limiting factors for grapevine distribution [8]. During heat stress, the ambient temperature rises above the threshold level of plant tolerance and, when extreme or long-lasting, may cause irreversible damage. Grapevines often encounter heat stress during the growing season that perturbs cell homeostasis, may affect proper development and fruit metabolism and consequently exert constraints on grape yield and quality. Although the grapevine has a good ability to adapt to various environmental pressures, long-lasting extremely high temperatures or heatwaves may permanently affect yield attributes and vine physiology [9]. Due to the upcoming climate changes, the knowledge of grapevine responses to heat stress is of particular importance for the sustainability of viticulture and one of the most important topics in grapevine biology.

Grapevines (*Vitis* spp.), like other plants, have an internal adaptive mechanism to combat heat stress. Metabolic processes such as respiration, photosynthesis and transpiration are very sensitive even in short-term temperature fluctuations. Photosynthesis is the most critical process in plants that is directly or indirectly affected by temperature. Heat stress, which is often accompanied by drought, may significantly reduce stomatal conductance and water-use efficiency. Recent progress in molecular biology has uncovered the major stress response pathways in plants and has broadened our view of abiotic stress responses and plant tolerance [10]. The availability of the complete grapevine genome sequence has provided an opportunity for the identification and characterization of various genes, cis-regulatory elements and trans-factors implicated in stress response [11]. After employed “omics” technologies, many abiotic heat-stress-inducible genes and proteins have been identified, although our current knowledge on the particular mechanisms and complex regulatory networks governing heat stress response and acquisition of tolerance in the grapevine is still far from complete. In this review, we summarize the current knowledge and recent progress in heat stress studies on the grapevine. Particular emphasis was given to the impact of heat stress on altering major phenological stages and implications in viticulture and on the physiological and molecular responses.

## 2. Global Warming Impacts on Grapevine Phenology and Viticulture

Climate change is expected to affect many aspects of the natural world, while its impact on agricultural production may be of particular importance. Viticulture is one of the sectors of agriculture to be affected by climate change and specifically global warming. Climate is the main factor limiting the geographical distribution of grapevines over the world and also a basic element of the so-called “terroir” concept, which is of great significance for the wine industry [12]. The primary impact of global warming in viticulture is on phenology, i.e., the timing of annually recurrent biological events, as grapevine sensitivity to heat stress is directly related to the phenological stages [13,14]. Grapevine phenology is thought to be one of the main natural indicators of heat stress and may be utilized to measure the effect of environmental changes on most sensitive developmental stages like flowering, veraison and grape ripening [13,15]. Many models have been created based on the tight link between temperature and phenology to predict the onset and duration of phenological stages in the near future [14]. This prediction is of extreme significance in arranging appropriate viticultural activities and winemaking choices [16].

**Figure 1:**
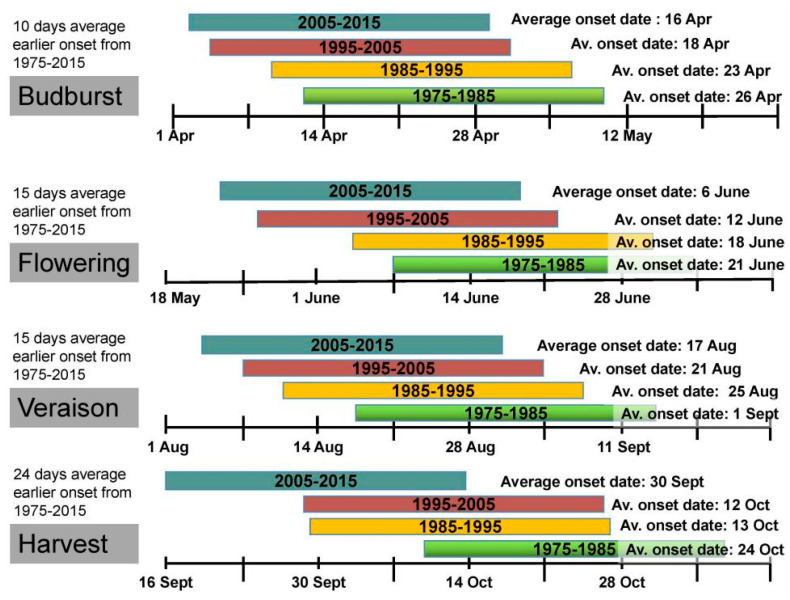
Phenological shifts based on average onset dates for bud break, flowering, veraison and harvest of four grapevine varieties (“Pinot Gris”, “Pinot Noir”, “Riesling” and “Muller Thurgau”) grown in Hainfeld (Southwest Germany) from 1975 to 2015. Based on data from a previously published study [18].

Among the most evident biological effects of global warming are the phenological shifts [5,17]. An analysis of data for four cultivars in south-west Germany, obtained from a previously published study [18], revealed an average 10–24-day shifts in the onset of most important grape phenological events from 1975 until 2015 (Figure 1). A significant advance is expected in most phenological stages, although the duration of each stage is depended on the sort of soil and the grape variety [3,15]. A direct interaction between the average temperature during the growing season and the duration of the annual vegetation cycle has been documented [3]. The effect of heat stress on the phenology of several grape varieties has been well investigated [19]. Shortening of the growth intervals and a prior onset of most phenological stages is likely to occur. Research based on these models anticipates the advancement of two to three weeks until 2050, while the phenomenon in the northern hemisphere vineyards will become more apparent [20,21]. According to Caffarra and Eccel [22], the most prominent phenological shifts refer to flowering and veraison rather than to budburst. Projection models indicate that higher temperatures may not shorten the period length from bud break to anthesis, but rather the length from anthesis until maturation will be shortened significantly [23].

Grape berry metabolism and juice quality are affected by both the onset and the duration of phenological stages [3]. In temperate zones of the northern hemisphere, the most suitable grape ripening period is usually in September, when the days are still warm and the nights are cool [20]. In such climates, early veraison dates would cause the berries to mature earlier under high-temperature conditions, which may have a negative impact on berry quality [14]. Early ripening results in the loss of wine typicity, changes in the aromatic character and loss of balance between sugar and acidity of the grape juice [21]. Grapes harvested earlier than expected thus result in wines with less organic acids, higher pH values, higher ethanol levels and altered sensory characteristics. In a future warmer climate, higher temperatures in wine-growing areas may also lead to the reduction of grape color due to the inhibition of anthocyanin biosynthesis [17,24].

Several wine-growing regions in southern Europe have already reached or even exceeded optimum thermal conditions for the currently cultivated varieties [25]. Fraga et al. [26] referred to recent elevated temperatures during grape berry maturation in the Iberian Peninsula, indicating a possible diminishing of wine quality in the near future. Likewise, higher temperatures during the growing season in Slovenia have resulted in a significant decrease in the total acidity content of early-ripening varieties [27]. The same changes have been also observed in many viticultural regions in Europe, like Germany and France [5,17], and in some viticultural regions in Australia, where recent studies have shown an earlier onset of most phenological stages and an overall shortening of the whole growing season period [28]. Global warming, however, will probably have the most serious impacts on the Mediterranean region, which is located in the middle of the tropical climate of North Africa and the temperate rainy climate of central Europe [26]. The Mediterranean basin is one of the largest wine-growing regions in the world, characterized by long growing seasons with moderate to warm temperatures. Throughout the year there is little seasonal change in temperatures, and winters are generally warmer than those of continental climates. As the suitability of several southern European wine-making regions will decline, due to global warming effects, the projected warming in central and northern European regions will result in prolonged frost-free periods and growing seasons that will favor wine quality [3]. Many studies have projected the possible extension of viticultural zones to new vine-growing regions in Europe showing that an increment of 4 °C in the mean annual temperature will bring a general shortening of the annual growth cycle [29]. An eastbound and northward move of viticultural territories is thus expected to incorporate England, Poland, Romania, Belarus and Ukraine. Another reasonable result of the temperature increase is expanding the areas suitable for viticulture to areas with higher altitudes, where the temperatures for vines are currently too low [22]. Besides the rise of the mean annual temperature, heatwaves during the crucial development stages of grape berry have increased in the last decades, and under enhanced global warming they are expected to worsen, becoming more frequent and more intense [30,31]. These prolonged periods of excessive heat events may have dramatic impacts on both the quality and the yield of grape production, despite the overall suitable weather conditions [13,31].

## 3. High-Temperature Effects on Grapevine Physiology and Berry Composition

### 3.1. Effects on Photosynthesis

Among the main physiological functions, photosynthesis is the first process to be directly affected by temperature variations [32,33]. It is reduced before other symptoms appear when the temperature rises above an optimum limit, which differs among species [34,35]. The optimum photosynthetic temperature for the grapevine is between 25 and 35 °C [36]. When the temperature is below 10 °C, most of the physiological processes decline, and at temperatures over 35 °C, heat acclimation mechanisms are activated [15,37]. Extremely high temperatures, i.e., above 40 °C, have drastic effects on photosynthesis mainly due to the disruption of the photosynthetic apparatus.

Field measurements conducted on the photosynthesis of grapevine leaves at a temperature range between 20 and 40 °C showed that compared with 25 °C the average photosynthetic rate (Pn) decreased with increasing temperature and was inhibited by 60% at 45 °C [38]. Several studies on grape leaves clearly show that Pn does not decrease significantly at 35 °C, but it is limited at a temperature of over 40 °C [34]. Greer and Weedon [38] suggested that the Pn reduction may be attributed to a 15%–30% reduction of stomatal conductance. This is probably because heat and drought stresses are tightly linked and reduced stomatal conductance may consequently increase the symptoms of heat stress as leaf temperature rises [39]. However, as is the case for other abiotic stresses, the effect of heat stress on stomatal conductance differs among grapevine varieties. For instance, “Touriga Nacional”, a Portuguese wine cultivar, keeps stomata open under mild heat stress, which is beneficial for the cooling of leaves via evaporation and thus may help keep photosynthesis unaffected [40].

**Figure 2:**
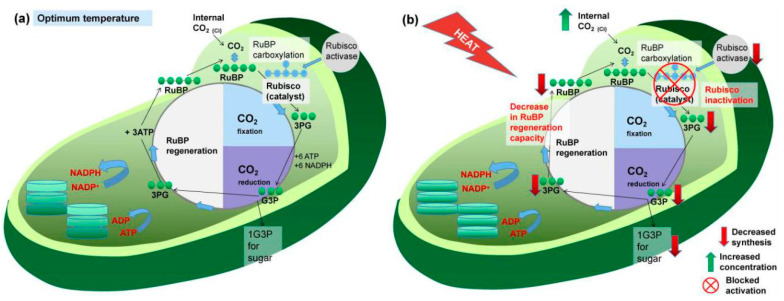
(**a**) Chloroplast function under optimum temperature; (**b**) disturbances of major biochemical processes of chloroplasts in grapevine leaves under heat stress. The activity of Rubisco activase is extremely heat-sensitive, and its inhibition blocks the activation of Rubisco and downstream reactions.

**Figure 3:**
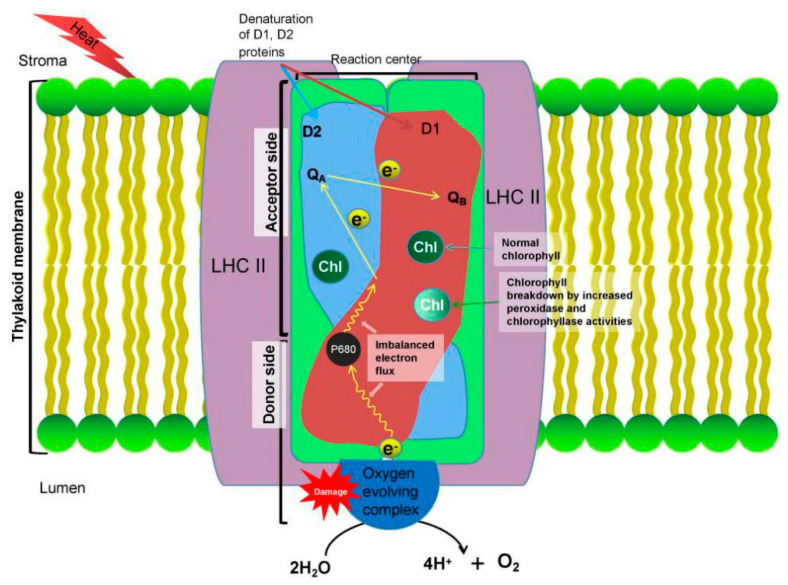
Photosystem II (PSII) is considered the most sensitive physiological system of the grapevine to heat stress. Extreme high temperatures cause dissociation to the oxygen-evolving complex (OEC), which results in the inhibition of the electron transportation from the OEC to the acceptor side of PSII. D1 and D2 are susceptible to heat inactivation, and under high-temperature regimes, chlorophyll degradation occurs due to the increased activities of peroxidase and chlorophyllase.

The decreased photosynthetic rate could also be attributed to the disturbances of biochemical processes, such as decreases in ribulose-1,5-bisphosphate (RuBP) regeneration capacity and ribulose bisphosphate carboxylase/oxygenase (Rubisco) activation [41], as is shown in Figure 2. Photosystem II (PSII) is considered to be the most sensitive physiological system of the grapevine to heat stress, usually suspended or destroyed before other cellular functions are disrupted [36,42]. It is formed by a complex of essential proteins, including D1 and D2, and is vital for the electron transfer during the photochemical stage of the photosynthetic pathway. Under high-temperature regimes, these core proteins are denatured and PSII impairment is observed after a few minutes to a few hours of heat exposure (Figure 3) [32,43]. High thermal stress even when applied at relatively short time periods, like 40 °C for 15 minutes, may cause serious and perhaps irreversible injury to the PSII of grapevine leaves [36]. As opposed to PSII, photosystem I (PSI) is relatively heat-stable.

There are generally two main factors that influence PSII sensitivity to high temperatures. The first is the increased fluidity of the thylakoid membrane, resulting in the disconnection of the PSII light-harvesting complex, and the second is the dependence of PSII integrity on electron dynamics [44]. In the grapevine, thylakoid membrane permeability is quite sensitive to heat stress [45]. A recent study showed that after heat treatments at 35 or 40 °C thylakoid membrane leakage, total chlorophyll content and chlorophyll fluorescence of “Cabernet Sauvignon” and *Vitis davidii* Foex. cv. Junzi vines did not change significantly, but the net photosynthetic rate was reduced. After heat treatment at 45 °C stress symptoms appeared with the fluidity of the thylakoid membrane increasing and total chlorophyll content decreasing [46]. It is noteworthy that prolonged high-temperature stress (e.g., a three-month period) mainly induced structural disorders of thylakoids. Chloroplasts in the mesophyll cells became round in shape, with smaller starch granules and more numerous plastoglobules than the control vines, indicating the beginning of senescence [45].

Damage to the thylakoid membranes is also associated with a decline in chlorophyll content [43]. Under high-temperature regimes, the chlorophyll-degrading peroxidase and chlorophyllase activities increase, resulting in a severe decline in chlorophyll content [47] (Figure 3). Decreased total chlorophyll content indicates inhibition of PSII. Thus, chlorophyll fluorescence measurements can be used to detect shifts in the photosynthetic machinery and as a good indicator for heat resistance in grape cultivars [48]. Strasser et al. [49] developed a method based on fluorescence transient analysis, namely the OJIP test, which explores changes in PSII photochemical performance and has been used as a measure of plant susceptibility to stress. This test can be applied to estimate many phenological and physiological expressions of PSII and is a unique method for in vivo examination of PSII behavior, including electron transportation and energy absorption [34,50]. The OJIP test may be used as a fast and simple method for measuring heat damage in grapes and can reveal information about the PSII electron transport chain. Xu et al. [51] conducted the OJIP test using different grapevine genotypes (“Riesling”, spine grape and “Jingxiu”) to investigate the response of the PSII electron transport chain to extreme heat stress (i.e., 47 °C). Results show that during the first 10 min of heat treatment the electron transport chain of PSII was highly sensitive to stress. High-temperature stress in grapevine may also cause serious damage to the oxygen-evolving complex (OEC) of PSII [52]. OEC participates in the splitting of water and the release of oxygen, resulting in an imbalance of the electron flux from the OEC toward the acceptor side of PSII [41] (Figure 3). The deactivation of the OEC is considered to be the cause of the reduced electron transport capacity caused by heat, especially at high temperatures. However, at moderate-high temperatures (e.g., 35°C) the damage in grapevine leaves is rather not significant, as leaves can easily alter the PSII properties to reduce OEC heat sensitivity [34].

The basic fluorescence (F0) and chlorophyll fluorescence, which is the ratio of variable fluorescence to maximum fluorescence (Fv/Fm), are parameters related to the tolerance of grapevine to heat stress [16]. However, it is not clear yet whether the inhibition of grape photosynthesis by high-temperature stress is due to a failure of electron transfer or to a reduction of Rubisco activity. Inactivation of Rubisco increases exponentially as temperature increases, and its activity drastically declines over 35 °C. Chlorophyll degradation due to heat stress can also reduce soluble protein content and change the speed of the Rubisco synthesis [16].

### 3.2. Effects on Transpiration

In the vineyard, heat stress is often accompanied by seasonal drought stress, which is a serious constraint in grapevine growth. Stomatal closure serves as the first-line defense from potential desiccation. However, transpiration is irreplaceable as part of the radiation energy is converted into latent heat through shifts in the opening of stomata [44]. Transpiration due to stomatal conductance is defined as the difference in intercellular and atmospheric water-vapor pressure divided by the total atmospheric pressure, which is often presented as a vapor pressure deficit (VPD) [53]. It is the main component of the energy balance of the leaves providing evaporative cooling to plants, necessary to keep leaf temperature below a maximum allowable limit [54]. Even a low transpiration rate can cause the leaf temperature to drop by a few degrees, which in some cases is the difference between growth and wilting. Average transpiration rates in grapevine leaves have been shown to increase five times almost linearly at a temperature range between 15 and 40 °C, i.e., from 0.5 to about 2.5 mmol m^−2^ s^−1^ [55]. However, further temperature rise up to 45 °C had no additional effect on the transpiration rate. In “Semillon” vines, the transpiration rate increased substantially with the increase in leaf temperature, particularly at high heat stress conditions (above 35 °C), which is consistent with the need for enhanced evaporative cooling [56]. Similarly, transpiration rates of “Chardonnay” increased four-fold as the temperature rose from 15 and 30 °C, and the rate was even higher at 35–40 °C, while in “Cabernet Sauvignon” the transpiration rates increased almost linearly with increasing temperature from 20 to 40 °C [57]. It has been suggested that “Semillon” vines exhibit relatively higher transpiration rates as compared to other international cultivars [58], thus its cooling capacity may keep the canopy temperature a few degrees lower than the air temperature.

Although temperature may affect grapevine stomatal conductance across the whole temperature range, there is only a small overall shift and not a direct link between temperature and stomatal conductance [59]. Stomatal conductance in most plants declines under high VPD levels, up to a given threshold. Accordingly, stomata of “Chardonnay” leaves did not respond to temperatures below 30 °C, but stomatal conductance declined strongly at elevated temperatures and high VPD values [55]. As opposed, “Shirah” vines when heat-stressed at normal VPD conditions exhibited 62% higher stomatal conductance than the control plants [60]. Taken together, it is the interaction of temperature with VPD levels that regulate stomatal conductance rather than the temperature alone. The differences recorded in stomatal responses to temperature changes and succeeding transpiration rates have led to the classification of vines into isohydric and anisohydric varieties, according to the sensitivity of their stomatal conductance to VPD variations. Differences between isohydric and anisohydric behaviors in stomatal response to VPD have direct effects on the heat stress tolerance of grapevines [60]. Anisohydric behavior may contribute to heat dissipation, given that the soil available water is sufficient to maintain transpiration, whereas reduction in stomatal conductance in isohydric varieties may enhance the damaging effects of high-temperature stress [61].

### 3.3. Effects on Grape Berry Composition

**Figure 4:**
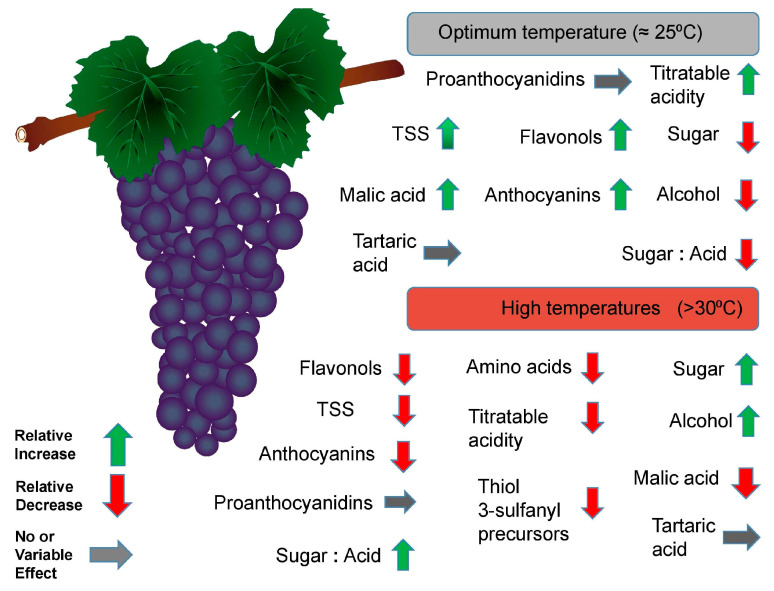
Summary of the effects of high-temperature stress on grape berry metabolism.

The chemical composition of grape berries is quite complex comprising several hundreds of different compounds, mainly water, fermentable sugars, organic acids, nitrogen compounds, minerals, pectins, phenolic compounds and aromatic compounds. Global warming is expected to change the temperature range in major viticultural areas, leading to changes in the composition of berries. The rate of grape berry metabolism strongly depends on the ambient temperature. Elevated temperatures perturb several metabolic pathways resulting in alterations in the biosynthesis of basic compounds that are critical for the grape must quality [62]. More specifically, the rise in temperature is expected to lower the acidity and increase the sugar content of berries, resulting in unbalanced wines with higher alcohol content and deprived of freshness and aromatic complexity [15,63]. Anthocyanin content is also reduced by high temperatures (Figure 4).

Due to the elevated sugar content of grapes in the last decades, the ethanol content of wines has increased accordingly. Many of the wines that used to have 11%–12% vol. ethanol in the 1980s now have about 13%–14% vol. [64]. Titratable acidity decreases as the temperature rises, being lower at 30 than at 20 °C [65]. The role of acidity in winemaking is extremely important, and the taste of wine is directly related to acid concentration. The main organic acids are tartaric acid and malic acid, which together make up about 80% of the total organic acid content. The acid harmony, defined as the relative concentration of tartaric to malic acid, along with the potassium content characterizes the acidity of grape berry juice. Under heat stress conditions, especially at the stage of grape maturity, the potassium concentration of berries increases, thereby increasing the pH value and finally reducing the total acidity [15]. As the temperature increases, malic acid is also metabolized faster than tartaric acid. The optimum temperature for malate accumulation is 20–25 °C, and a dramatic decline is observed above 40 °C [66]. However, the biochemical and molecular mechanisms by which malate degradation enhances at high temperatures and the way downstream metabolic pathways are affected are poorly explored [67]. When the temperature is relatively high during the day, low night temperature is essential to ensure a low pH. This is quite important for the sustainability of the grape/wine sector considering that global warming due to climate change is expected to be associated with elevated temperatures at night rather than during the day. High temperatures also affect the ratio of sugar-acid balance. Elevated temperatures can promote the accumulation of sugars and the concomitant degradation of organic acids, with the acidity being more drastically affected than the sugars. This results in lower acidity for the same sugar content in grapes grown under warmer conditions (Figure 4).

Heat stress restrains the formation of anthocyanins and flavor compounds in grapes grown in temperate regions [65,68]. For most common varieties, the ideal temperature during the grape maturation stage for the optimum formation of aroma compounds is within the range of 20 and 22 °C [62]. Reductions in color formation are observed when the temperature exceeds 30 °C, and at temperatures over 37 °C decreased grape color and increased volatilization of aroma compounds are observed [15,64]. The main components of the grape color are anthocyanins, mainly found in the grape skins of red grapes. Under high-temperature conditions, reductions of delphinidins, anthocyanins, petunidins and peonidin-based anthocyanins in grapes were observed but not in the biosynthesis of malvidin derivatives [15]. Anthocyanins, like other phenolic compounds, are also highly unstable and susceptible to thermal degradation. It is worth mentioning that the combination of heat and drought stress has less effect on the degradation of anthocyanins and sugars than heat stress alone, and this is because water deficiency may alleviate the deleterious effects of high temperature in the degradation of these compounds [69].

## 4. Molecular Responses to Heat Stress

Plants, like other living organisms, have the ability to perceive various abiotic stress signals from the environment through specialized sensor molecules and receptors and to consequently activate signaling pathways as a response to these stimuli. The identification of sensors for stress signals and the elucidation of the downstream signaling cascades are fundamental in plant science and big questions in grapevine biology. It has been shown that different receptors may perceive various types of stress signals [39]. However, there are both unique and overlapping abiotic stress signals and there is much functional redundancy in genes encoding stress sensor proteins. High temperature may act as a sole stress factor in plants, but in the grapevine, as in other summer crops, heat is often combined with drought stress. Thus, plants have developed adaptive molecular mechanisms to counteract the detrimental effects of these combined constraints. Most of the known protein stress sensor molecules are located in the plasma membrane and include members of diverse gene groups or families, like receptor-like kinases (RLKs), G protein-coupled receptors (GPCRs), histidine kinases (HKs), ABA receptors and calcium sensors [70,71].

**Figure 5:**
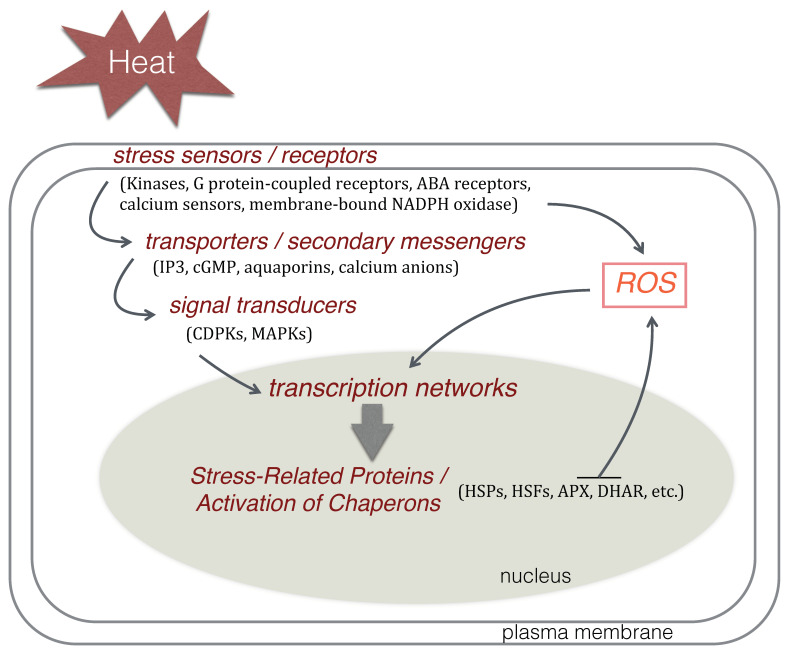
A simplified scheme for the heat-stress signal transduction pathway focusing on major regulatory components and stress-responsive genes identified so far in the grapevine. Protein stress sensor molecules perceive heat-stress signals and transmit them through secondary messengers initiating transcription networks to provide stress tolerance. Abbreviations: IP3, inositol 1,4,5-trisphosphate; cGMP, 3′,5′-cyclic guanosine monophosphate; CDPKs, calcium-dependent protein kinases; MAPKs, mitogen-activated protein kinases; HSPs, heat shock proteins; HSFs, heat stress transcription factors; APX, ascorbate peroxidase; DHAR, dehydroascorbate reductase; ROS, reactive oxygen species.

The perceived signals are then transmitted through secondary messengers, such as lipids like IP3, cyclic GMP, aquaporins and especially Ca^2+^. During high-temperature stress, Ca^2+^-binding proteins such as calmodulin (CaM), CaM-related proteins, Ca^2+^-dependent protein kinases (CDPK) and calcineurin B-like protein (CBL) perceive the elevated Ca^2+^ concentration and initiate transcription networks and the activation of protein kinases (PKs), such as the mitogen-activated kinases (MAPKs). PKs in turn phosphorylate specific transcription factors (TFs) and induce stress-response genes that may act as molecular chaperones to provide heat and/or drought tolerance [71,72,73], as shown in Figure 5. Heat shock proteins (HSPs) and reactive oxygen species (ROS) scavenging enzymes are known targets of heat-stress-responsive TFs and play crucial roles in the adaptation of plants to heat stress [74].

Following the characterization of the first *Arabidopsis* protein kinase receptor in the early 1990s, a number of different PKs have been identified in various plant species that regulate downstream signaling pathways [71,75]. Apart from their involvement in stress sensing and subsequent response, PKs may also play critical roles in various developmental processes. Computational identification and classification of the entire collection of protein kinases in the grapevine genome (also referred to as kinome) have revealed a high number of genes (approximately 1200) grouped into 121 gene families. Among them, the calcium-dependent protein kinases (CDPKs) and the MAPKs are involved in a variety of developmental processes and also in grapevine responses to various types of stress [76]. Transcriptomics of heat-stressed grapevine leaves revealed upregulation of genes encoding components of calcium- or calmodulin-mediated signal pathways, including calmodulin and CDPKs, which induced the expression of heat stress transcription factors (HSFs) and heat shock proteins (HSPs), pointing to the implication of Ca^2+^-mediated signals in grapevine heat stress response [77].

Both HSPs and HSFs are central players in the heat stress response and the acquisition of thermotolerance in plants [74,78]. HSPs are molecular chaperones that accumulate under heat stress to prevent misfolding and aggregation of proteins and to facilitate protein refolding under conditions of denaturing stress [79]. HSPs may be induced under various other abiotic stresses, including low temperature, oxidative stress, osmotic stress and desiccation, and also have roles in plant development and disease resistance [80,81,82]. There are five HSP families in plants, namely HSP100, HSP90, HSP70, the chaperonins HSP60 and the small HSP (sHSP) family [83]. Among them, the 90-kDa HSP90 family differs from most other molecular chaperones in that their known client proteins are signal transduction proteins, like transcription factors and kinases [84,85]. The *Vitis vinifera* HSP90 family has been identified and, in accordance with *Arabidopsis,* it comprises four cytoplasmic and three organelle-specific members [86]. Transcriptional analysis of VvHSP90 genes in various vegetative tissues and under different high-temperature stresses (30, 37, and 45 °C) uncovered the role of VvHSP90.1 as a bona fide heat-inducible gene. VvHSP90.1 was also differentially regulated with respect to the severity of the heat stress, suggesting that the HSP90-mediated grapevine heat stress response not only senses shifts in temperature but may also monitor the magnitude of the stress [86]. Various other members of the HSP family were shown to be upregulated during heat stress, and their transcripts declined dramatically after recovery, like the HSP101, HSP21 and HSP22, suggesting that some sHSPs may have important roles in the heat tolerance of grapevines [77]. The authors also showed the upregulation of ascorbate peroxidase (APX) and dehydroascorbate reductase (DHAR) genes under heat stress, suggesting an important role of these enzymes in grape leaves to scavenge ROS generated by heat stress. Besides toxic by-products, ROS may also act as signaling molecules, thus their concentration in the cell should be precisely controlled [87]. ROS in the cell respond early under heat stress conditions and induce the expression of HSFs and the accumulation of HSPs [88]. As expected, some members of the HSF family in the grapevine, like the HSF7 that promote the expression of HSPs, exhibited increased expression levels under heat stress and were downregulated after the subsequent recovery period. In addition, genes of the calcium- and calmodulin-mediated transduction pathways were also heat-induced or recovery-regulated, pointing to a prominent role of Ca^2+^-mediated signals in the heat stress response of the grapevine. Interestingly, the upregulation of some RLK genes, which are also induced by wounding, pathogen attack and salicylic acid, points to overlapping pathways with other biotic, abiotic and ABA stress signaling pathways in grapevine leaves [77]. Extensive overlapping signaling pathways of heat stress with other stresses may explain the high percentage of grapevine genes (68% of the assembled genes) detected under high-temperature conditions (35–45 °C) through a high-throughput transcriptomic analysis [89]. Furthermore, after integrating transcriptomics and proteomics data, the authors verified previous results showing that HSPs are the main components of the heat tolerance machinery in grape, along with some important transcription factors such as Multiprotein Bridging Factor 1c and Heat Shock Transcription Factor A2.

## 5. Conclusions

Climate change has led to a significant advancement in the grapevine phenological stages over the last decades. If the annual temperature continues to rise and the global warming phenomenon to amplify, as climate models predict, worldwide viticulture is going to face a real threat in the near future. Estimating the magnitude of future risk will help to develop rational and sustainable strategic approaches for grape growers [90]. Even though some local varieties may be adapted to such environmental conditions, most international cultivars that originate from cooler climates will probably not withstand such extreme heat-stress conditions [7,91]. The local grapevine germplasm from regions of warm and dry climate may serve as an alternative. Exploration and agronomic evaluation of the indigenous diversity for heat-stress-tolerant varieties or clones would be of particular importance for the sustainability of viticulture and the wine industry [25,92].

In addition, the ongoing research in grape physiology coupled with molecular biology data mainly acquired from “omics” approaches have uncovered a number of stress-responsive factors and molecular regulators with prominent implications to heat stress tolerance. Although the heat response mechanisms of the grapevine share many similarities with other crops and the model plant *Arabidopsis*, in particular, the grapevine as a perennial fruit crop species displays several distinct features, as it has been also shown in the case of the HSP90 family [86]. Therefore, it is important to identify the particular components of grape regulatory networks governing heat stress response and acquisition of tolerance. There is no doubt that the biggest challenge would be the application of this scientific knowledge in the vineyard, e.g., in breeding programs aiming to develop genotypes tolerant to environmental stresses. It is anticipated that the forthcoming advances in metabolomics and systems biology will further accelerate the development of stress-tolerant clones, so as to achieve sustainable viticulture.
